# Using a comprehensive audit to identify local context prior to care bundle design and implementation for inadvertent perioperative hypothermia in colorectal surgery

**DOI:** 10.1136/bmjoq-2020-001132

**Published:** 2021-01-13

**Authors:** Judith Tanner, Stephen Timmons, Roger Bayston, Kimberley Adams, Bryn Baxendale

**Affiliations:** 1 School of Health Sciences, University of Nottingham Faculty of Medicine and Health Sciences, Nottingham, UK; 2 Nottingham University Business School, University of Nottingham, Nottingham, UK; 3 School of Medicine, University of Nottingham Faculty of Medicine and Health Sciences, Nottingham, UK; 4 Colorectal and Stoma Care, Nottingham University Hospitals NHS Trust, Nottingham, UK; 5 Trent Simulation & Clinical Skills Centre, Nottingham University Hospitals NHS Trust, Nottingham, Notts, UK

**Keywords:** implementation science, patient safety, surgery

## Abstract

**Background:**

The first step in bundle design or implementation is to identify the problem being addressed. Several validated approaches are recommended to facilitate this. These include using systematic reviews, adverse event triggers and risk assessment tools. However, these methods do not fully take the local context into account, which will limit the effectiveness of the bundle.

**Aim:**

This study explores the potential benefit of using a comprehensive audit to identify an organisation’s local context prior to designing and implementing a care bundle.

**Method:**

A comprehensive audit comprising observations of four patient journeys, interviews with 21 staff and clinical data was carried out at one large National Health Service trust in England. A patient warming care bundle was used as the exemplar.

**Findings:**

Each of the three data collection methods identified specific local practices which would be addressed within the planning and implementation stages of a care bundle. These practices would not have been identified through other recommended methods.

**Conclusion:**

A comprehensive audit, comprising observations, interviews and clinical data is a successful method to identify local contextual issues prior to care bundle implementation.

## Background

Some care bundles have made minimal improvement on patient outcomes.^1^ Even bundles which have been highly effective in one setting have not achieved similar levels of success in other settings.^2–4^ Researchers suggest this is due to deficiencies in the implementation of the bundle rather than problems with the bundle itself.^5^ One reason for a bundle’s limited success may be failing to understand the local context prior to implementation.^6^ The first step in designing or implementing a care bundle is to identify the problem.^7^ Understanding the local context does not appear to be a routine feature of bundle design. In a systematic review of bundle implementation in critical care, only 8 out of 99 papers conducted a local needs assessment.^8^

Several validated approaches are proposed to identify the problem. These include adverse event trigger tools, the model for improvement, root cause analysis, systematic reviews, failure mode and effects analysis risk assessment tools, criteria tools such as Appraisal of Guidelines for Research and Evaluation (AGREE II), checklists, and morbidity and mortality meetings.^7 9 10^ However, these approaches may not identify fully local contextual factors which will limit the effectiveness of a bundle.

This paper describes how undertaking a different approach, a comprehensive audit, can inform both the design and the implementation of a care bundle by identifying an organisation’s situational context. A surgical patient warming care bundle is used as an exemplar.

In 2016, the National Institute for Health and Care Excellence (NICE) Inadvertent Perioperative Hypothermia guideline was updated.^11^ The aim of this guideline is to prevent surgical patients from becoming hypothermic (core temperature less than 36°C) during their surgical journey. Perioperative hypothermia is caused by several factors including increased exposure to cold surroundings, interventions with cooling side effects such as skin preparation solutions or washouts, plus the administration of anaesthesia which redistributes blood flow.^12^ Hypothermia results in complications including surgical site infection, myocardial infarction, increased perioperative blood loss, increased length of stay in recovery, increased length of hospital stay and delayed wound healing.^11^

Following the publication of the updated guideline, we (a group of clinicians and researchers) wished to implement a surgical patient warming care bundle to maximise warming interventions with the aim of reducing patient complications, primarily surgical site infection.

However, consideration of the validated approaches led us to believe that these methods would not sufficiently incorporate the local context and instead we decided to undertake a comprehensive audit. This would provide an understanding of the situational context of the factors affecting patient temperature which would influence the design and implementation of our warming bundle.

## Aim

The aim of this study was to explore the potential benefit of using a comprehensive audit to identify an organisation’s local context which would inform designing and implementing a care bundle.

## Methods

The audit was carried out at one large National Health Service trust in England from October 2016 to January 2017. There were three components to the audit to give a full and comprehensive understanding from which a care bundle could be designed and implemented, and progress measured. These were;

1. Observations of the patient journey—including the generation of a process map.
2. Qualitative interviews with staff—to identify factors which may influence bundle implementation.
3. Clinical data—to identify compliance with bundle (warming) interventions, and clinical outcome measurements (temperature).

Observations were carried out first to identify staff groups to invite for interview.

### Setting

The study focused on colorectal surgery as this specialism should provide a rich seam of data regarding warming interventions. Patients having open or laparoscopic colorectal surgery are at high risk of developing hypothermia as surgery may require access via an extensive open wound, procedures are lengthy, lasting around one and half hours for open surgery and two and a half hours for laparoscopic surgery, and they are carried out under general anaesthesia. These events exacerbate heat loss and intraoperative warming interventions recommended by the NICE are therefore routinely applied.^11^ At the trust where the audit was carried out there is one surgical ward predominantly dedicated to colorectal surgery patients and two operating theatres allocated for colorectal surgery. All patients having surgery within the colorectal theatres under the care of the colorectal surgical teams were included in this study.

### Observations

Patients were followed through their elective surgical journey, from the admissions ward to arrival on a postsurgical ward, to generate a process map. Four patient journeys were considered sufficient to generate the process map.

One patient was selected for follow through on each of 4 days. Selection was pragmatic and depended on the availability of the observer, however, each patient was typical of patients having colorectal surgery. Field note data collected included the departments through which the patients travelled with entry and exit times, the professional group status of the staff who treated them and the interventions, events or settings which affected their body temperature. There was no patient interaction, and no personal details were obtained. One researcher analysed the data to create a process map and this was checked by a second researcher.

### Interviews

Staff, purposively invited to take part in the study, represented the professional groups from the clinical areas identified through the process map and thus were all involved in the patient warming process. These were nurses who worked in the admissions wards, surgical wards, operating theatres, anaesthetic rooms or recovery units, plus operating department practitioners, surgeons and anaesthetists. Ten interviews comprising at least two staff from each professional group and clinical area were considered sufficient to give an insight into the staff contribution to bundle interventions. Posters were placed in staff rooms within these departments inviting staff to take part in an interview.

The first three interviews were used as pilot interviews and reviewed before the remaining interviews took place. Data from the pilot interviews were included with the data from the main interviews. The interviews were semistructured. Questions were open ended, generated by the care bundle development team and focused on role, contribution towards patient warming and the warming journey. The interviews lasted around 20 min, took place on site and, with written consent, were audio digitally recorded. Interview data was transcribed and analysed thematically. One researcher coded the data into themes which were checked by a second researcher. Participants were not invited to feedback on the findings.

### Clinical data

Data from all 124 patients having surgery within the colorectal theatres and operated on by the colorectal surgical teams during a 2-month period was collected from patients’ electronic notes. Data focused on compliance with warming guidelines and included surgical procedure, temperature and the application of active body warming devices. Anonymised data were entered onto a spreadsheet. Data were presented using simple descriptive statistics.

### Researcher characteristics

Patient observations and staff interviews were carried out by a female operating department practitioner with a qualification in research methods. The researcher previously worked with some of the staff interviewees but none of the patients.

### Patient and public involvement

We did not involve patients or the public in the design, conduct or reporting of this study because of limited time scales and resources. This is a limitation of the study as this would have been beneficial.

## Findings

### Observations

**Figure 1:**
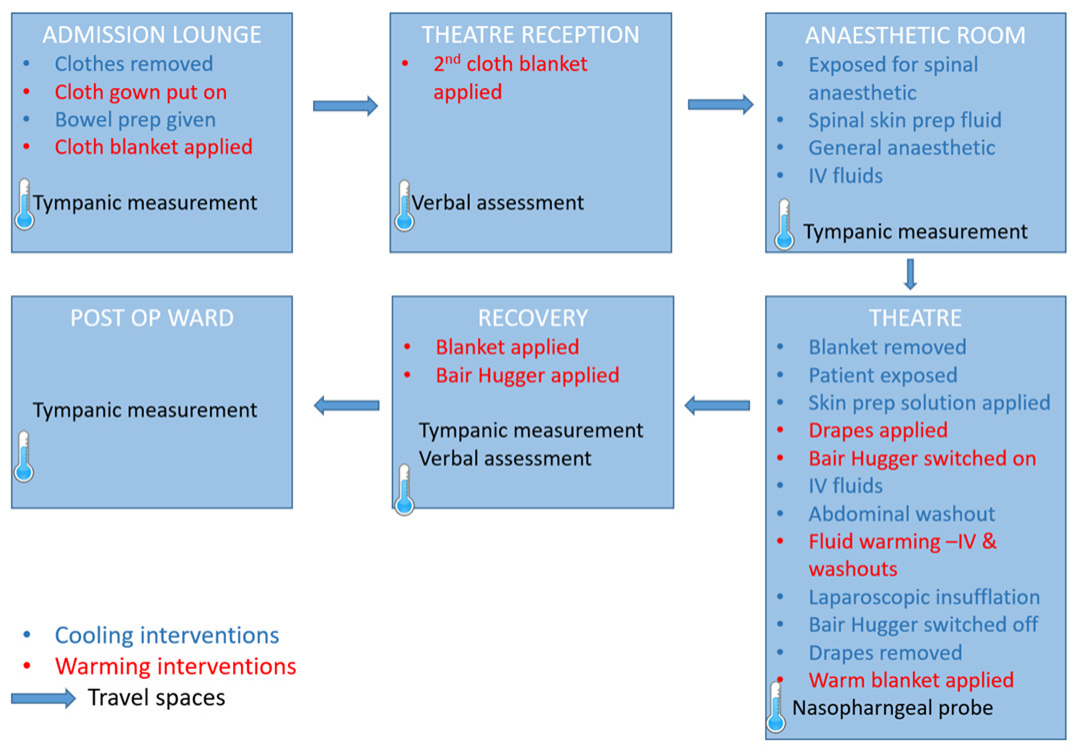
Process map.

Observation data from the four patient follow-throughs were compiled to produce a process map (figure 1) showing the spaces through which the patients progressed. Six ‘fixed spaces’ enclosed by physical boundaries (eg, wards or recovery bays) were identified and there were also ‘travel spaces’, such as corridors and lifts, between the fixed spaces. During the journey, patients came into contact with four professional staff groups who contributed to warming practices. These were operating department practitioners, surgeons, anaesthetists and nurses who worked in the admissions wards, surgical wards, operating theatres, anaesthetic rooms or recovery units. In addition, non-registered care support workers, theatre reception staff, patient escorts and porters also contributed to warming, or cooling, activities.

During the journey, temperature was recorded in four of the six fixed spaces using either tympanic or nasopharyngeal measuring devices. Various interventions were implemented within each fixed space which either deliberately warmed or inadvertently cooled patients. These interventions and their associated resources were specific to each fixed space. For example, anaesthesia (which reduces patients’ temperature) was given in the anaesthetic room and forced air warming blankets (Bair Hugger) were given in the operating theatre.

The time taken for the four patients to complete the surgical journey from admissions lounge to arrival on the postsurgical ward was 12 hours and 46 min, 13 hours and 55 min, 8 hour and 15 min, and 12 hours and 14 min. The long length of the overall journey was surprising to the clinicians on the research team and to other clinicians with whom we discussed this informally.

### Interviews

Twenty-one interviews were conducted with four admissions nurses, three theatre nurses, two operating department practitioners, four recovery nurses, three ward nurses, two surgeons and three anaesthetists. No participants dropped out of the study. No interviews were conducted with non-registered staff which is a limitation of this study. The recordings were transcribed and grouped into the following four broad themes; ‘boundaries’, ‘professional knowledge and guidelines’, ‘responsibility’ and ‘local beliefs and practices’. ‘Equipment’ was a sub theme within ‘boundaries’.

#### Boundaries

When staff spoke about departments and units, they described them as having unique identities, practices and functions. For example, ‘Theatres are cold’. ‘The receiving area is cold’, ‘They do [warming] in the theatre’, ‘That happens in the admissions lounge’.

In addition, each space was identified as having its own resources. For example, different warming resources with varying levels of effectiveness were available within each space. The admissions unit had the most basic equipment,

I often draw the curtains across, if it’s draughty. (Admissions Nurse 1).

I’ve heard of something like that [Bair Hugger™ forced air warming blanket]. I mean obviously we don’t use them here. And when we take the patients to theatre reception they’ve got blankets that are in a warmer. (Admissions Nurse 3)

Theatre reception and the anaesthetic room had slightly better equipment (warmed blankets) than the admissions ward, but this was still limited.

I think our receiving area is a cold area. We have just the warm blankets from the cabinet in reception and in the anaesthetic room (Theatre Nurse 3)

The most advanced warming equipment was found in the operating theatre and the recovery unit.

In surgery we put the Bair Hugger on them, we heat all their fluids (Theatre Nurse 1).

Well in recovery we are very lucky. We’ve got blanket warmers and also we can use our Bair Huggers, so that’s really good. (Recovery Nurse 1).

In the ward, the final space on the patient journey, only warm blankets were available. Different types of temperature measuring devices were also used in different spaces.

In addition to the fixed spaces such as wards and departments, gaps between the spaces were identified. These ‘travel spaces’ included corridors and lifts where patients were transported between departments. These spaces were quite important to the warming journey as warming, or more likely, cooling, activities occurred within them, yet as they were ‘unstaffed’ areas, no-one took responsibility for these travel spaces

I also think that like there are really simple ways to try and stop cooling pre-op, like getting the patient to walk to theatre rather than sticking them in a wheelchair when they don’t need a wheelchair. (Recovery Nurse 2).

I think some of the emergency cases and trauma cases coming down off the ward struggle a bit with temperature, because they’ve often got cold, especially coming up through emergency department…That can be quite a problem… (Anaesthetist 3).

Boundaries existed between the spaces and resources were not routinely shared across these boundaries.

We don’t keep a Bair Hugger on the ward, but we can try and borrow it and take it back as soon as possible. (Ward Nurse 3)

We have been known to ring down to get one of the Bair Hugger and see if we can borrow one, which we have borrowed before on night shifts to warm a patient up (Ward Nurse 2)

In addition to resources not moving across boundaries, staff, with the exception of surgeons and anaesthetists, rarely moved across boundaries. This meant that many staff were unaware what happened in other stages of the patient journey and did not have an overview of the whole surgical process.

I’m not aware of them [NICE Hypothermia Guidelines] but I’m sure it is an important role in the theatre. (Admissions Nurse 1)

So actual Bair Huggers …I know they’re used in theatre but I don’t know what they do (Admissions Nurse 2).

Only as far as them having a warm blanket…I don’t know if anything else is offered to them (Theatre Nurse 1).

I think because we don’t really see the patients who then come back and have antibiotics …we’re not really getting a proper picture of what’s actually happening (Operating Department Practitioner 1).

#### Equipment

In addition to some warming and measurement equipment only being available within certain clinical areas and not always being shared, there were also some concerns about the usability or reliability of equipment.

We sometimes have some issues with the equipment, say that it doesn’t work all the time. (Anaesthetist 1)

Unfortunately we are using a different thermometer and I hear a lot of complaints about it. I feel the patient is warm but when I measure the patient’s temperature using tympanic, it gives me an unbelievably low temperature. … [Medical equipment service unit] are very good in returning and looking at the equipment and sending it back but I think we really have to look at the reliability of the equipment we are using. (Recovery Nurse 1)

#### Professional knowledge and guidelines

The observations showed that at least four professional staff groups, plus several non-registered staff, contributed to the warming or cooling of patients. Knowledge regarding warming and its effect on clinical outcomes appeared to be influenced by profession. Anaesthetists and surgeons had the most knowledge with admissions nurses perhaps being least aware. Although anaesthetists were the group most likely to be aware of, and have read, the NICE warming guidelines and recommendations, they were not fully compliant with them.

I don’t think we probably fully adhere to it because I think there are issues with temperature measuring beforehand … (Anaesthetist 1)

No. I suspect we don’t monitor patients’ temperature as frequently as could be the case, and I don’t think we make use of active warming in as many cases as we would be indicated on the guidelines… (Anaesthetist 3)

I think it would be impractical [to adhere to the NICE hypothermia guidelines] to delay the start of surgery if their temperature was less than 36… (Anaesthetist 2)

#### Responsibility

Staff were not entirely clear who, if anyone, was responsible for keeping the patient warm.

In [recovery] it’s us as individual recovery nurses. I would say in theatre it’s the team as a whole I guess, the anaesthetist predominantly but I think it’s everyone’s sort of role isn’t it, if they notice… (Recovery Nurse 4)

I guess it’s the anaesthetist who is doing most of the monitoring. And I think the surgeon would expect the anaesthetist to be having a close eye on it. So it’s the anaesthetist when they’re in theatre and it’s probably us when they’re here. It’s kind of whoever’s leading on their care at that point in their experience. (Recovery Nurse 2).

#### Local beliefs and practices

There was a commonly held view that patients who were undergoing surgical procedures which were perceived to have a ‘short’ duration did not require temperature monitoring or active warming with a Bair Hugger.

So we will make a decision to measure temperature based really on whether we’re going to do active warming and whether it’s a long procedure (Anaesthetist 1).

…if it’s a short procedure then we don’t measure the temperature in theatres (Theatre Nurse 1).

If the perceived operation duration is short, I wouldn’t measure temperature… Because it is unlikely that you will realistically be able to do anything about it (Anaesthetist 2).

I think it should probably be selective because some operations, if they’re short operations, don’t really need it do they? (Surgeon 1)

### Clinical data

Clinical data (table 1) showed temperature measurement was routinely taken in the admissions ward, the recovery unit and the surgical ward. Staff interviews and observations support the clinical data, showing that temperature was rarely measured in the theatre reception or the anaesthetic room. Documentation was mixed in the operating room. Only 2% of patients who had their temperature recorded on the admissions ward were hypothermic and only 2% of patients who had their temperature recorded on the post op surgical ward were hypothermic. However, around one third of patients who had their temperature recorded in the anaesthetic room, the operating room and the recovery unit were hypothermic. Compliance with active warming in the operating room was good, with 96% of hypothermic patients given a forced air warming blanket (Bair Hugger), but less so in the recovery unit (47%).

**Table 1:** Temperature measurement and active forced air warming

	Admissions ward	Theatre reception	Anaesthetic room	Operating room	Recovery unit	Surgical ward
Patients with temperature recorded	115/124 (93%)	0/124 (0%)	9/124 (7%)	64/124 (51%)	114/124 (91%)	98/124 (79%)
Patients recorded as hypothermic	2/115 (2%)	–	3/9 (33%)	25/64 (39%)	36/114 (32%)	2/98 (2%)
Patients given forced air warming	0	0	0	78/124 (63%)	19/124 (15%)	0
Hypothermic patients given forced air warming	0	/	0	24/25 (96%)	17/36 (47%)	/

Theatre staff identified some operations as having a short duration and this influenced whether patients having these procedures had their temperature measured or active warming applied. Short operations were not formally defined, but there was a shared understanding among the theatre staff interviewed. Short operations included procedures such as examinations under anaesthesia, pilonidal sinuses, colonoscopies, biopsies and botox injections.

**Figure 2:**
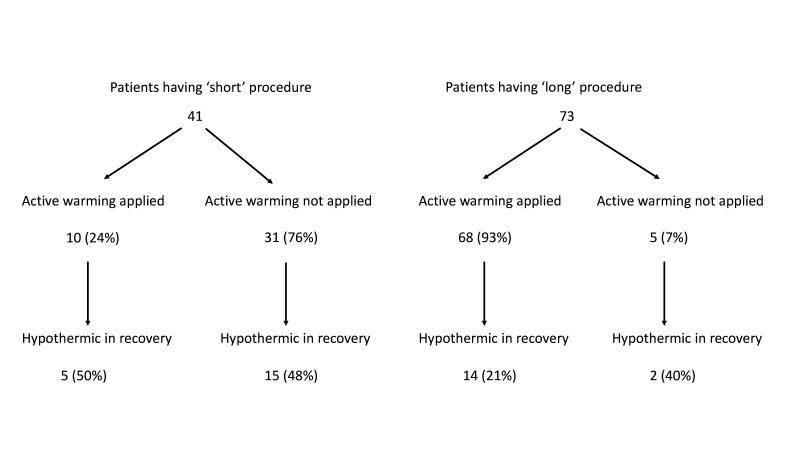
Perceived operation duration.

Clinical data showed that patients having ‘short’ operations were less likely to be given active forced air warming, compared with patients having ‘long’ operations; 24% vs 93% (figure 2). Despite having ‘short’ operations, 48% of these patients who were not given active warming were hypothermic on admission to the recovery unit. Although they were perceived as short, the average length of time from start of anaesthesia until admission to the recovery unit for a ‘short’ procedure was 1 hour 16 mins. The average duration for a ‘long’ procedure from start of anaesthesia until admission to the recovery unit was 4 hours 0 mins.

## Discussion

The aim of this paper was to explore the potential benefit of using a comprehensive audit to identify the local context of an organisation prior to designing and implementing a care bundle.

Through using observations, interviews and clinical data and with a patient warming care bundle as the exemplar, we identified the following events and phenomena: key staff and departments, boundary issues, responsibility, guideline adherence and local practices.

When papers report on studies planning and designing care bundles to prevent surgical site infection or inadvertent perioperative hypothermia, staff selected to contribute to the process are usually limited to professional staff from the operating theatre (surgeons, anaesthetists and operating room staff).^13^ However, looking at the patient journey as a whole system has highlighted the complexity of patient warming and has identified ‘overlooked’ staff and departments which contribute to this process. This included registered and non-registered staff from the admissions lounge, theatre reception and the wards. Using observations to build the patient journey will enable us to include all relevant staff and departments in care bundle planning meetings.

Perhaps the most overlooked individual who was at the centre of the patient observations was the patient. Patients feel cold before their core temperature registers a reading less than 36°C. This is a stressful and unpleasant experience for patients. If patients were included as stakeholders in care bundles to prevent inadvertent perioperative hypothermia then outcome measures would likely include verbal assessments in addition to clinical indicators.

Boundaries existed around each of the six departments which were formed by geography, and also by differences in practices and access to resources. Practices and resources were not shared across boundaries which meant that temperature monitoring and warming was not applied consistently throughout the patient journey. A further boundary existed between the different staff groups who contributed to patient warming as information and knowledge appeared to vary by group. This is a concern as geographical and professional boundaries are known barriers to implementation and can create risk areas where patient care can be compromised.^14 15^ Interventions are required to reduce boundaries and financial input or reorganisation may be necessary to have access to resources in each department.

Lack of clarity regarding patient responsibility was an issue, especially for the ‘travel’ spaces. This is important as accountability and responsibility are associated with increased compliance.^16^ This can be addressed in care bundle implementation by making roles and responsibilities explicit during the planning and implementation meetings and subsequent training days.

Clinical data showed that at least 30% of patients were hypothermic on admission to the recovery unit and compliance with some national guideline recommendations was poor when staff found them impractical. An English national patient warming survey found similar findings.^17^ We, therefore, need to further explore guidelines that are considered to be impractical as these are unlikely ever to implemented, and engage staff with impartial feedback data which is shared across all contributing professional and non-registered staff from all departments.^18^

Not monitoring and not warming patients who were undergoing what were perceived to be short operations was a specific local cultural issue which we only identified through the interviews and clinical data. This practice may have arisen through a misinterpretation of an NICE recommendation which states that active warming should be applied for patients having anaesthesia for more than 30 min.^11^ This practice will need to be addressed locally through education and possibly supported with ‘nudges’.^19^ For example, warming prompts could be triggered within operating theatre software where activities such as duration of care is recorded.

While we did not explore in depth the usability, usefulness and use of the equipment and devices used to monitor and manage patient temperature, there were clearly concerns about these. Not addressing these concerns would likely limit the successful compliance with any warming bundle which relied on their involvement.^20^

The combination of observations, interviews and clinical data contributed to identifying local contextual issues which need to be addressed in the design and implementation of a care bundle to maximise its effectiveness. As no adverse events or root cause analyses had been triggered and systematic reviews and guidelines do not mention the specific issues mentioned here it is unlikely that these issues would have been identified through conventional approaches used to develop care bundles.

## Conclusion

An assessment of the local setting prior to the implementation of a care bundle is necessary to facilitate the implementation of the bundle and maximise its success. We have shown that a comprehensive audit, comprising observations, interviews and clinical data is a successful method to identify local contextual issues and is therefore essential in the implementation of any national guideline.
